# Cytoplasmic Capes Are Nuclear Envelope Intrusions That Are Enriched in Endosomal Proteins and Depend upon β_H_-Spectrin and Annexin B9

**DOI:** 10.1371/journal.pone.0093680

**Published:** 2014-04-04

**Authors:** Juan Wu, Katelyn J. Bakerink, Meagan E. Evangelista, Graham H. Thomas

**Affiliations:** 1 Departments of Biology and of Biochemistry and Molecular Biology, The Pennsylvania State University, University Park, Pennsylvania, United States of America; 2 School of Public Health, Harvard Medical School, Boston, Massachusetts, United States of America; University of Toronto, Canada

## Abstract

It is increasingly recognized that non-erythroid spectrins have roles remote from the plasma membrane, notably in endomembrane trafficking. The large spectrin isoform, β_H_, partners with Annexin B9 to modulate endosomal processing of internalized proteins. This modulation is focused on the early endosome through multivesicular body steps of endocytic processing and loss of either protein appears to cause a traffic jam before removal of ubiquitin at the multivesicular body. We previously reported that β_H_/Annexin B9 influenced EGF receptor signaling. While investigating this effect we noticed that mSptiz, the membrane bound precursor of the secreted EGF receptor ligand sSpitz, is located in striking intrusions of the nuclear membrane. Here we characterize these structures and identify them as ‘cytoplasmic capes’, which were previously identified in old ultrastructural studies and probably coincide with recently recognized sites of non-nuclear-pore RNA export. We show that cytoplasmic capes contain multiple endosomal markers and that their existence is dependent upon β_H_ and Annexin B9. Diminution of these structures does not lead to a change in mSpitz processing. These results extend the endosomal influence of β_H_ and its partner Annexin B9 to this unusual compartment at the nuclear envelope.

## Introduction

The spectrin based membrane skeleton (SBMS) is best known as the structural element, which gives shape and strength to the vertebrate erythrocyte [1]. However, it is increasingly recognized that the SBMS components have roles in endomembrane trafficking and protein recycling. Here spectrin provides an anchor for the dynactin complex [2] and variously modulates secretory [3], [4] and endocytic pathways [5], [6], [7] by as-yet-undetermined mechanisms. Spectrins also have nuclear roles in association with Fanconia's Anemia proteins in the repair of interstrand crosslinks [8] and in nuclear positioning [7]. Here again the precise role of spectrin remains enigmatic and any relationship to its cytoplasmic roles is undetermined.

β_H_-spectrin modulates the endosomal pathway in *Drosophila*, where it partners with AnxB9 to regulate protein movement to the multivesicular body [5], [7]. Part of the evidence for this assertion comes from a genetic interaction between loss-of-function β_H_-spectrin mutations or AnxB9^RNAi^ with the *rhomboid^veinlet^ (rho^ve^)* mutation. Specifically, *rho^ve^* is suppressed by both these genetic elements and introduction of loss-of-function alleles in core endocytic and multivesicular body (MVB) functions tied this phenotype to the endosomal pathway [5]. It is well established that the EGF receptor (EGFR) is endocytosed and either recycled or degraded *via* well characterized endo-lysosomal compartments [9]; however, recent data has suggested that the membrane-bound EGFR ligand precursor mSpitz also passes through an endosomal compartment to be cleaved by the *rhomboid* protease prior to secretion [10]. Using wing vein formation as a developmental assay, we previously proposed that the interaction between *rho^ve^*, β_H_-spectrin/AnxB9, and endosomal loss-of-function mutations might arise from elevated EGFR signaling due to a traffic jam at the MVB – trapping the EGFR/ligand complex in signaling endosomes [5]. However, the observation that processing of mSpitz, the precursor of the mature secreted sSpitz, can occur in an endosomal compartment [10] suggests that it is also possible that loss-of-function β_H_-spectrin mutations and AnxB9^RNAi^ might instead be increasing ligand processing.

While investigating the connection between β_H_/AnxB9 and sSpitz production we noticed that mSpitz not only resides in a perinuclear ER compartment as previously reported, but that it accumulates to its highest levels in structures that intrude on the nucleus. Our investigation of these structures reveals them to be cytoplasmic capes – infoldings of the nuclear membrane that are associated with perinuclear vesicle accumulation. We find that the capes appear to be enriched in endocytic recycling compartments and ubiquitylated proteins, and that the number of such capes is greatly reduced or eliminated in β_H_^RNAi^ and AnxB9^RNAi^ glands. To begin to investigate the relationship between β_H_/AnxB9, EGFR signaling and cytoplasmic capes, we directly demonstrate that EGFR signaling is elevated upon β_H_ knockdown, but that mSpitz processing is not elevated. In contrast, EGFR can be induced to accumulate in cytoplasmic vesicles that label with β_H_-spectrin. These results identify cytoplasmic capes as potential sites of protein sorting that contain at least one cargo protein, mSpitz.

## Materials and Methods

### Fly strains

Oregon-R or the transformation host *yellow white* were used as wild-type lines. UAS-β_H_^RNAi^ and UAS-AnxB9^RNAi^ lines were described in [5]. Driver lines AB1-Gal4 (salivary gland), engrailed-Gal4 (posterior compartments) and MS1096-Gal4 (wing blade) as well as UAS-Rab6::YFP, UAS-Rab4::YFP, UAS-Rab7::YFP, UAS-Rab10::YFP, UAS-RabX1::YFP, UAS-GFP::myc::2xFYVE, and UAS-EGFR were obtained from the Bloomington stock center (Bloomington, IN; #1824, #30564, #8860, #23251, #23269, #23641, #24097, #23274, #42712 and #5368 respectively). UAS-mSpitz::GFP was gift from Dr. Eyal Schejter (Weitzman Institute, Rehovot, Israel). UAS-BicD::GFP was gift from Dr. Simon Bullock (MRC-LMB, Cambridge, England).

### Antibodies and Immunostaining

Antibodies used in this study are as follows: Mouse monoclonal anti-dpErk (1∶50, Sigma-Aldrich, St. Louis, MO); Mouse anti-Ubiquitin (1∶1000; Enzo Life Sciences, Plymouth Meeting, PA); Affinity purified Rabbit anti-β_H_ #243 (1∶10; [11]); Guinea pig anti-Hrs (1∶800) and guinea pig anti-EPS15 (1∶500; both from Dr. Hugo Bellen, Baylor College of Medicine, Houston, TX); Rabbit anti-Rab 5 (1∶75; from Dr. Marcos Gonzalez-Gaitan, University of Geneva, Geneva, Switzerland). Rabbit anti-Lava Lamp (1∶5000; from J. Sisson, University of Texas, Austin, TX). The monoclonal antibody anti-lamin C (LC28.26) developed by Klaus Weber was obtained from the Developmental Studies Hybridoma Bank developed under the auspices of the NICHD and maintained by The University of Iowa (Iowa City, IA); All secondary antibodies for immunofluorescence were from goat, labeled with Alexafluor dyes and were obtained from Invitrogen (Carlsbad, CA). Secondary antibodies were used at [1∶250] following preabsorbtion against fixed wild type embryos. Texas Red X-conjugated wheat germ agglutinin was purchased from Molecular Probes (Eugene, OR).

To stain for dpERK in wing imaginal discs, 3rd instar larvae that were actively crawling on the wall of vials were dissected in 1xPBS and immediately transferred into 4% PFA in 1xPEM (1 mM MgSO_4_, 2 mM EGTA, 100 mM Pipes.HCl pH 6.95) at room temperature. After 10 minutes of dissection, collected wing discs were transferred to an orbital shaker for 25 minutes at room temperature. After fixation, wing discs were washed with PBS, blocked and extracted with Incubation Solution 1 (10% normal goat serum, 0.2% Saponin, 0.3% deoxycholate, 0.3% Triton X100 in PBS) for 1 hr at room temperature. All the subsequent incubations used the Incubation Solution 2 (10% normal goat serum, 0.1% Triton X100 in PBS). Primary and secondary antibody incubations were done overnight at 4°C. For this antigen discs were imaged immediately after staining as the signal fades quickly.

3^rd^ instar salivary glands were dissected as rapidly as possible and held in ice cold PBS until fixation on ice in 4% w/v paraformaldehyde in PEM on ice for 60 minutes with gentle agitation. Glands were rinsed in ice-cold PBS prior to blocking and extraction in Incubation Solution 1. All subsequent antibody incubations and washing was done in Incubation Solution 1. For some antigens (Ubiquitin and Hrs) antibody penetration to the depth of the nucleus was facilitated by a post-fixation crosscut in middle-distal region of the gland.

Samples were imaged on a CARV II spinning disc confocal (BD Biosystems, Rockville, MD) with a Retiga EXi camera (Q Imaging systems, Surrey, BC) and iVision 4.0 software (Biovision, Exton PA).

### Electron Microscopy

Salivary gland samples for serial block face scanning electron microscopy (SBF-SEM) were dissected and accumulated in ice cold PBS. Initial fixation was performed overnight in 2.5% glutaraldehyde, 2% formaldehyde, 0.15 M cacodylate buffer pH 7.4, 2 mM CaCl_2_ at 0°C. Following three 5 min rinses in 0.1 M cacodylate buffer pH 7.4 glands were postfixed in 0.1% tannic acid, 0.1 M cacodylate buffer pH 7.4 at room temperature, washed as before and treated for 1 hr in 2% OsO_4_, 41 mM potassium ferrocyanide, 0.2 M sodium cacodylate at 4°C. Following three 5 min washes in dH_2_O the glands were incubated for 20 min in 1% thiocarbonhydrazide at 60°C. Following three 5 min washes in dH_2_O the glands were incubated for 30 min 2% OsO_4_ in dH_2_O at room temperature, rinsed again in dH_2_O as before and stained overnight in 1% uranyl acetate at 4°C. Following three 5 min washes in dH_2_O the glands were incubated for 30 min in 20 mM lead nitrate, 30 mM potassium aspartate at 60°C. Following three final rinses in dH_2_O the glands were dehydrated through an ethanol series, equilibrated in acetone and embedded in Epon 812 resin according to standard protocols. SBF-SEM was performed by GATAN using the GATAN 3 view system (Gatan Inc., Pleasanton, CA) installed in a FEI Quanta 600 F scanning electron microsocope (FEI, Hillsboro, OR). Sections were taken every 50 nm. 2,900 sections covered ∼20 cells in each wild-type and mutant gland. For standard transmission electron microsopy, glands were prepared as described in Phillips and Thomas (2006) and imaged using either a JEM 1200 EXII, (JEOL Peabody, MA) or an Tecnai G2 Spirit BioTwin (FEI, Hillsboro, OR) transmission electron microscope. The latter was also used for or electron tomography.

Final processing and figure assembly for this paper was done using Adobe CS4 (Adobe, San Jose, CA). Image series from SBF-SEM were assembled into movies using Final Cut Express (Apple, Cupertino, CA).

## Results

### The EGFR ligand precursor mSpitz is found in a nucleus-associated compartment

**Figure 1:**
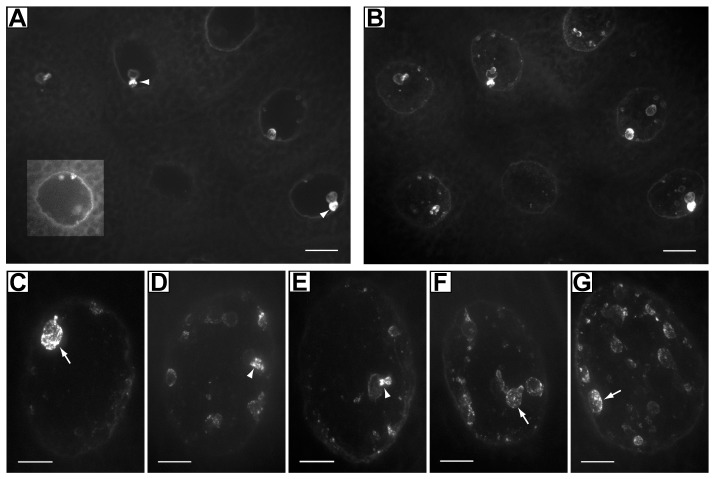
Expression of mSpitz::GFP in third instar salivary glands. **A,B** A low magnification view showing that the capes are found in every nucleus. Panel A shows the appearance in a single confocal section. The images are dominated by the intranuclear concentrations of mSpi::GFP so the region around one nucleus has been enhanced to allow the perinuclear distribution to become visible. Panel B is a maximum projection of the same group of nuclei. **C–G** – A high magnification view of five individual nuclei, exemplifying the range of cape size and morphology seen. All images are maximum projections of confocal sections across each nucleus. Scale bar represents 20 μm in A,B, 10 μm in C–G.

**Figure 2:**
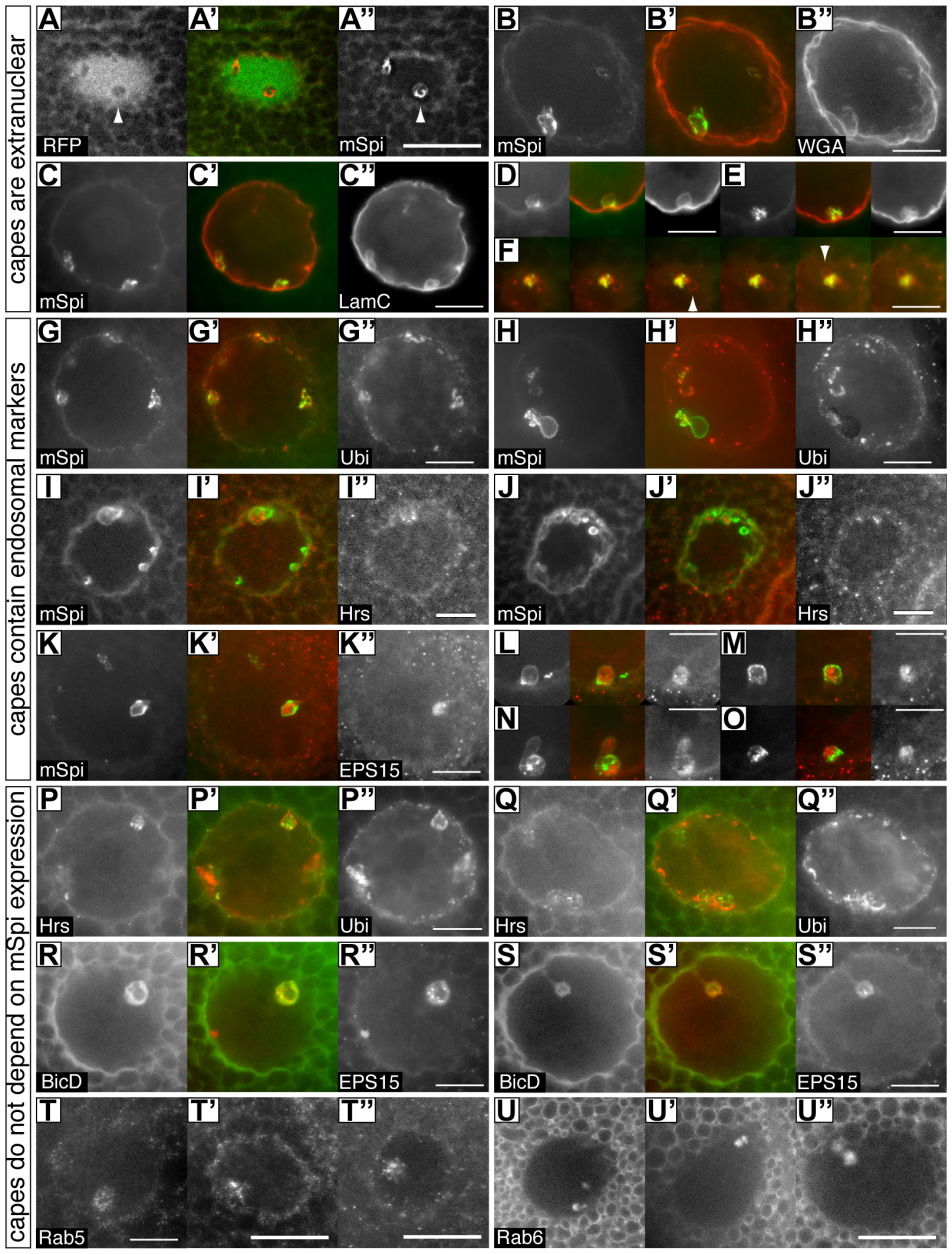
Cytoplasmic capes contain ubiquitylated proteins and endosomal markers and are found in wild-type glands. Each set of three images shows one nucleus or cape from a salivary gland showing the distribution of two proteins. The center panel is a merged red/green image with the left panel in green. Due to the varied morphology of each cape multiple examples are shown for most markers. A–P express mSPI::GFP and costaining is for the indicated proteins: **A-A”** – Nucleoplasmic RFP is excluded from capes (arrowheads). This image is a single plane from a nucleus where the image stack had been deconvolved; **B-B”** – Wheat germ agglutinin (WGA) stains glycosylated proteins of the nuclear envelope and outlines the capes indicating that they are infoldings of this membrane and that the contents are therefore extranuclear. **C–F** – Lamin C similarly coats the cape membranes. D,E show individual capes from other nuclei. Panel F shows a series of confocal images taken at 0.5 μm intervals through a cape at the top of a nucleus. Chambers associated with individual capes where mSpitz is very low or absent are often seen with this marker (arrowheads). **G–H”** – Ubiquitin (Ubi) puncta are found in most capes at their periphery (see also P–Q”). H shows a saggital view. **I–J”** – Occasional Hrs puncta are found in the central space of the terminal chamber; **K–O** – EPS15 puncta are found in most capes in the central space. L–O show individual capes from other nuclei. Panels P–U” do not express mSpi::GFP demonstrating that capes are present in wild-type glands. Costainings are as indicated: **P–Q”** – Ubiquitin (Ubi) puncta surround Hrs puncta; **R–S”** – BicD::GFP is not obviously punctate and fills the central space containing EPS15 puncta. Note the large central vesicle that excludes both markers in example R. **T-T”** – Rab5 puncta are found in the central space; **U-U”** – Rab6 is not obviously punctate and fills the central space. Several other Rab proteins do not enter the capes. Due to the widely varying extent and morphology of each cape, some panels show single confocal planes whereas others are maximum projections of up to 8 planes taken at 1 μm intervals. All scale bars represent 10 μm except for panels A”, T’, T” and U” which are 20 μm.

While investigating the effects of β_H_ or AnxB9 knockdown upon EGFR signaling, we expressed mSpitz::GFP, a fusion of GFP to the membrane bound precursor of the secreted EGFR ligand sSpitz [12], in 3^rd^ instar salivary glands (AB1-Gal4>UAS-mSpitz::GFP). As expected mSpitz::GFP accumulates strongly in a perinuclear region previously identified as endoplasmic reticulum (Figure 1; [12], [13]). However, we also noticed striking amounts of the protein that appeared to be concentrated inside the nuclear perimeter in large structures with connections to the nuclear periphery (Figure 1). The presence of mSpitz::GFP in similar structures was previously seen, but went unremarked ([14] Figures 2G, 2N, S1A; [15] Figure 1A; [16] Figure 1E). These structures seem to be substantially bigger in the large, highly polytenized nuclei of the salivary gland. Subsequent ultrastructural analysis (see below) indicates that these structures are the same as ‘cytoplasmic capes’ [17] and so this term will be used hereinafter.

Two lines of evidence indicate that these structures are not nucleoplasmic. First, coexpression of NLS-RFP with mSpitz::GFP shows that each cape excludes the RFP signal (Figure 2A). Second, two different markers for the nuclear lamina and membrane show staining around the cape periphery (Figure 2B–F). Together these data indicate that the capes represent involutions of the nuclear membrane.

The number and size of the capes varies from nucleus to nucleus as does their detailed morphology but each exhibits two general regions: A large rounded terminal chamber connected by an often narrower region to the nuclear periphery (arrows in Figure 1C,F,G). mSpitz::GFP is present at the membrane of the terminal chamber where it is often punctate, and also seems to be concentrated in the neck region (arrowheads in Figure 1A,D,E), possibly due to the amount of convoluted membrane present in this region (see below). mSpitz::GFP appears to be concentrated in the capes in comparison to the rest of the perinuclear ER.

### Ultrastructural analysis of cytoplasmic capes

**Figure 3:**
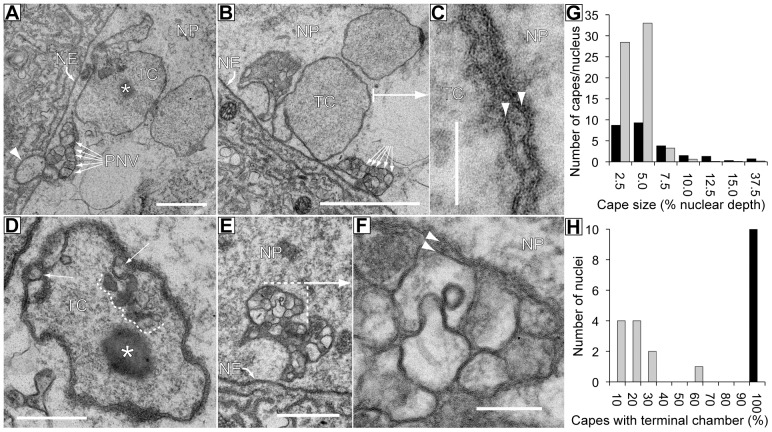
Ultrastructural analysis of cytoplasmic capes. TEM images from three capes in wild-type nuclei. **A, B** – These images shows two capes where regions of perinuclear vesicles (PNV) and the terminal chamber (TC) are visible. NE – nuclear envelope. NP – nucleoplasm. As illustrated in A the PNV region is often associated with an out folding of the outer nuclear membrane (arrowhead), non-membrane bound granules (asterisk) and small vesicles (see D). Scale bars are 1 μm. **C** – Higher magnification view of part of the membrane from a terminal chamber in B (arrow) showing two membrane bilayers (arrowheads). Scale bar is 200 nm. **D**–A terminal chamber containing several vesicular and granular organelles (dashed line, asterisk). Two PNV are also visible at the periphery of this chamber (arrows). **E**–A second cape showing only the region of PNV. Scale bar is 2 μm. **F** – Higher magnification view of several PNV in C (dashed line with arrow). PNV are bounded by a single bilayer and are separated from the NP by a single bilayer and often protrude into one another. See also serial sections in Movie S1. **G** – Chart showing the number of capes per nucleus with a given size (arbitrarily estimated in the Z dimension in serial block face SEM image series as the number of 50 nm sections from first appearance to disappearance in serial sections and normalized to the nuclear diameter in the same direction; see Movie S2). Data are shown for wild-type (black bars) and AnxB9^RNAi^ (grey bars). Many more small capes are present when AnxB9 is knocked down. **H** - Chart showing the number of fully sectioned nuclei containing capes with a terminal chamber. Data are shown for wild-type (black bars) and AnxB9^RNAi^ (grey bars). Whereas all capes end in a terminal chamber in wild-type, few do when AnxB9 is knocked down.

To further understand the nature of the capes, we performed an ultrastructural analysis of a wild-type salivary gland (i.e. not expressing mSpitz::GFP) by serial block face SEM through about 20 nuclei. Only one type of structure is seen that matches the size and geometry of the capes imaged by immunofluorescence (Figure 3). The capes appear to be infoldings of the nuclear envelope with no conspicuous ‘lumenal’ space evident until the most interior region, where they open out into a large chromatin-free space that is delimited by a double membrane (Figure 3 and Movies S1, S2). Within this space we see occasional single-membrane bound vesicles as well as non-membrane bound granules (Figure 3A,D and Movie S3). The regions proximal to the nuclear boundary contains a large number of single membrane-bound vesicles separated from the nucleoplasm by an additional single membrane indicating that these are vesicles in the perinuclear space as previously described [17]. These regions are often associated with out-foldings of the outer nuclear membrane. The overall size of the structure ranges from 0.4 to 6.5 μm (95% are ≤2.5 μm), while the number of capes per nucleus ranged from 6 to 71 with the bulk being 5% or less of a nuclear diameter (Figure 3E). The number of capes seen at the ultrastructural level is much higher than the number seen by immunofluorescence, so our original identification of these structures was based upon those that turn out to be the largest present. Presumably many of the smaller puncta at the nuclear membrane in our immunofluorescence images represent this smaller population (Figure 1).

### Cytoplasmic capes contain ubiquitylated proteins and endosomal markers

Previously, when investigating the relationship between β_H_-spectrin (β_H_), AnxB9 and endosomes, we had noticed the concentration of ubiquitylated proteins in similar structures (see Figure 5 in 5). Coupled with the knowledge that processing of secreted sSpitz is completed in an endosomal compartment [10], we therefore chose to further investigate the nature of the cytoplasmic capes by staining for endosomal markers. Several markers of the endosomal system are often or always found in capes (Figure 2G–U). The perinuclear region is enriched in puncta containing ubiquitylated proteins that are particularly concentrated at the periphery of the cape membranes (Figure 2G,H,P,Q). In addition, we detected variable levels of the endosomal markers EPS15, Hrs (ESCRT 0) and Rab5 as puncta in the central space of these structures (Figure 2I–O, R–U). EPS15 is frequently present, Hrs and Rab5 less so. In addition, Rab6::YFP and BicD appear to freely enter the central space (Figure 2R,S,U). However, there is selectivity in cape content, as several other compartment makers are not found in the capes (Figure S1).

Capes are readily detected with these markers in the absence of mSpitz::GFP expression indicating that their presence is not an artifact of overexpressing this protein (Figure 2P–U). Within the capes overlap between many of the markers is not precise suggesting that they contain a complex mix of compartments. This concentration of endosomal/MVB-related proteins, coupled with the concentration of ubiquitylated proteins and the mSpitz::GFP cargo suggests that the capes represent a region of protein sorting and processing. The presence of membrane bound vesicles in electron micrographs of the terminal chambers (Figure 3A,D and Movie S3) coupled with the punctate staining of endosomal markers seen in Figure 2, suggests that many of these may be endosomal organelles captured by the in-folding of the nuclear envelope.

### Cytoplasmic capes depend upon β_H_ and AnxB9 but do not appear to affect EGFR signaling

**Figure 4:**
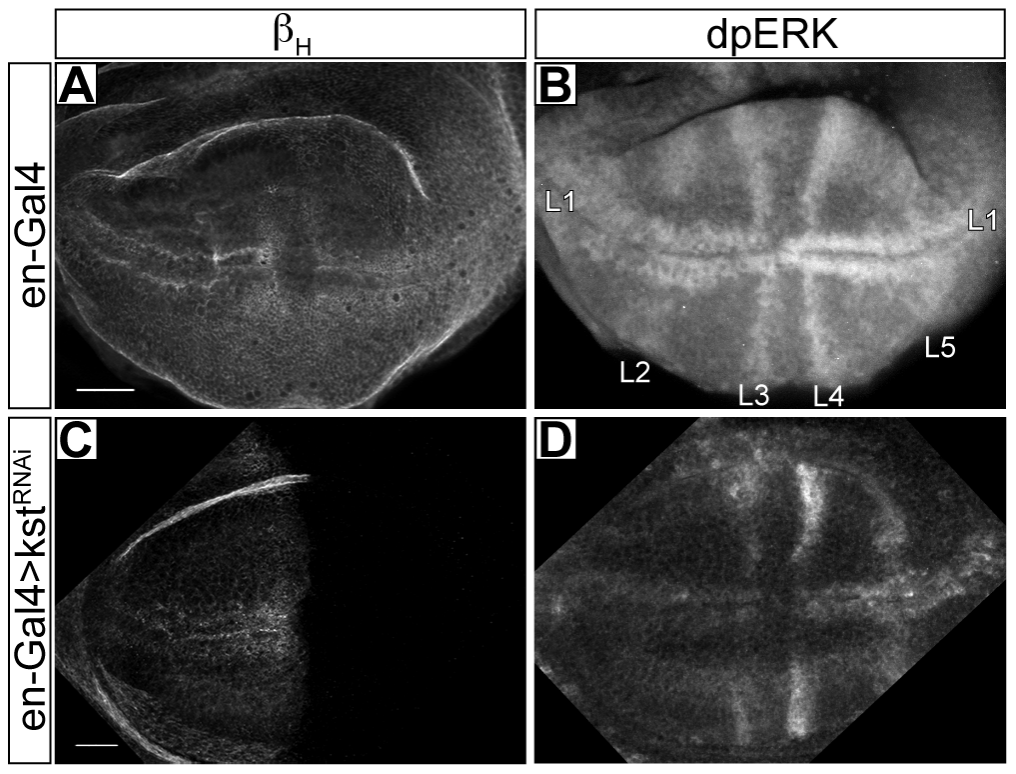
dpERK levels are elevated when β_H_ is reduced. **A**- A wild-type wing imaginal disc stained for β_H_. **B**- The same disc stained for activated MAP kinase (dpERK). Staining is most apparent at the anlagen of veins L1 (wing margin) and L3–5 as indicated. The intensities in the two compartments are roughly equal. **C**- An engrailed-Gal4>β_H_^RNAi^ disc stained for β_H_, which is no longer detectable in the posterior compartment. **D**- The same disc stained for dpERK. Staining is elevated in the posterior half of L1, while L4 and L5 are conspicuously more intense. Experiments were done at 25^O^C. Scale bar represents 20 μm.

We wondered if the presence of mSpitz in the capes was functionally important for EGFR signaling. A reduction in β_H_ or AnxB9 results in disruption of the endosomal system and was postulated to result in an elevation in EGFR signaling [5], providing an opportunity to probe the role of capes in this pathway. To more directly confirm this result we stained for activated MAP Kinase (MAPK) using an anti-dpERK antibody in 3^rd^ instar wing imaginal discs where β_H_ or AnxB9 had been reduced in the posterior compartment using the engrailed-Gal4 driver (en-Gal4). In the wild-type discs β_H_ expression is ubiquitous (Figure 4A), and elevated levels of dpERK staining mark the anlagen for each wing vein (Figure 4B; [18]). When β_H_ levels are significantly reduced in the posterior compartment (en-Gal4>β_H_^RNAi^; Figure 4C) there is elevated staining for dpERK in the veins of that compartment (Figure 4D) indicating that EGFR signaling is indeed increased upon β_H_ reduction. Quantitation of the fluorescence intensity on either side of the anterior-posterior boundary along the wing margin shows a significant increase in the posterior/anterior ratio from 0.88±0.14 (N = 8) in driver only discs to 1.61±0.31 (N = 25) in en-Gal4>β_H_^RNAi^ discs (P≤0.0002, Students T test [heteroscedastic]). We conclude that β_H_ knockdown indeed results in increased EGFR signaling.

**Figure 5:**
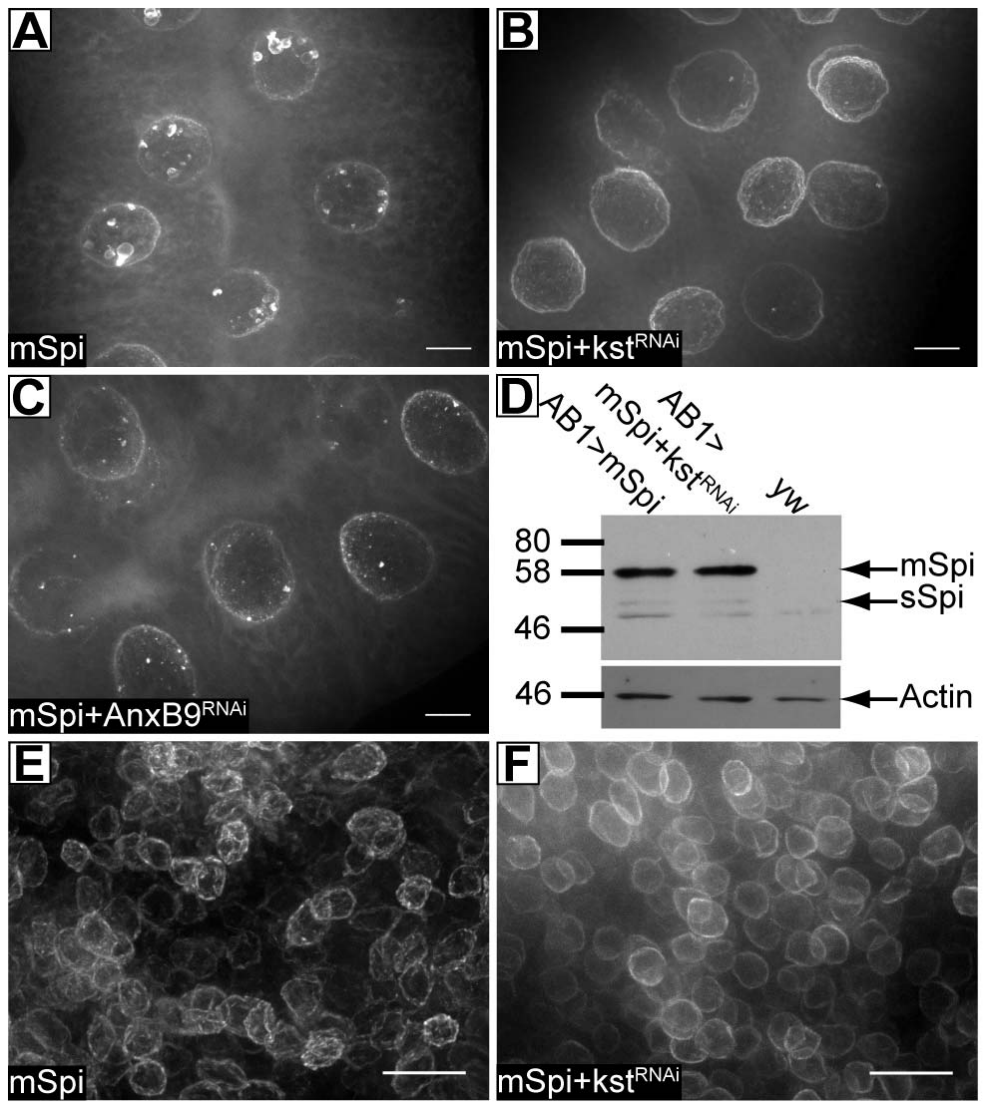
Cytoplasmic capes are dependent upon β_H_ and AnxB9, but sSpitz production is not affected by β_H_ knockdown. **A** – A wild-type salivary gland expressing mSpitz::GFP (AB1>mSpitz::GFP); **B,C** – Glands expressing mSpitz::GFP where β_H_ was knocked down (AB1>mSpitz::GFP+kst^RNAi^). Just the occasional cape is seen at this resolution. **D** – Immunoblot on dissected salivary glands expressing mSpitz::GFP with or without β_H_ knockdown. Five pairs of glands are loaded for each genotype. In the upper panel the blot was probed with an anti-GFP antibody. mSpitz::GFP runs at about 58 kDa and the cleaved ligand, sSpi at 50kDa. The lowest band is non-specific. The lower panel is the same blot probed for Actin as a loading control. The amount of processed sSpi is not to be affected by the loss of the capes. **E** – The wing pouch region from a wild-type wing disc (MS1096>mSpitz::GFP). mSpitz::GFP marked nuclear envelopes have a rough, slightly punctate appearance. **F** – The wing pouch region from a wing disc where β_H_ was knocked down (MS1096>mSpitz::GFP+kst^RNAi^). mSpitz::GFP now smoothly coats the nuclei. All images are maximum projections of multiple confocal sections. Scale bars represent 10 μm (E–F) or 20 μm (A–C).

β_H_ and its partner AnxB9 have roles in endosomal maturation through the MVB stage [5], so we next tested to see if cape morphology or number is affected by reducing these proteins. Knockdown of either β_H_ or its partner AnxB9 dramatically reduces the number of capes detectable by immunofluorescence, leaving only the occasional small structure (Figure 5B, C). The loss of capes was not due to Gal4 dilution upon introduction of the knockdown constructs because co-expression of mSpitz::GFP with UAS-NLS-RFP did not similarly eliminate them (Figure 2A). To determine if this is a salivary gland-specific phenomenon, mSpitz::GFP was also imaged in the wing disc (MS1096 Gal4>mSpitz::GFP). Here, as in the examples in the literature, capes are present but are quite small giving a roughened texture to the perinuclear staining (Figure 5E). The number of capes was again substantially reduced in the absence of β_H_ in this tissue (Figure 5F). These results suggest that the presence of mSpitz::GFP in capes is a general phenomenon and that the size of this compartment is tissue specific.

The disappearance of the capes when β_H_ or AnxB9 is knocked down is a specific effect because neither reduction in the levels of AnxB11 (AB1>*anxB11^RNAi^*), nor overexpression of wild-type and dominant negative EGFR, full length β_H_ (AB1>EP-*kst*), an internally deleted variant of β_H_ (AB1>minikarst; [5]) or segment 33 of β_H_ (AB1>βH33;[19], [20]) perturbs the size and number of the capes (not shown).

We also examined an AnxB9 knockdown gland by serial block face SEM. In this gland the frequency of the smallest type of cape is greatly elevated but none have a terminal chamber (Figure 3E,F). Thus there is a good correspondence between our observations by immunofluorescence and the presence/absence of the larger capes in the electron microscope. We still see some small dense membrane infoldings at the nuclear envelope and speculate that these represent abortive attempts to form capes in the absence of AnxB9.

To investigate the relationship between the cytoplasmic capes and EGFR signaling we examined the response of ligand precursor production and receptor distribution to changes in β_H_ level. Immunoblot analysis indicated that the overall levels of mSpitz::GFP did not change, nor did the amount of processed sSpitz (Figure 5D) suggesting that the disappearance of capes does not have a major effect on this process. Reduction of β_H_ (AB1-Gal4>β_H_^RNAi^) did not cause a major change in EGFR distribution (compare Figure S2A and B), but overexpression of EGFR results in receptor internalization and accumulation in an EPS15 and β_H_-associated endosomal compartment (Figure S1C, D). These data are consistent with a close relationship between β_H_ and the EGFR rather than with mSpitz processing, and are consistent with our published model: That β_H_ remains associated with internalized cargo vesicles until released by AnxB9, and that elevated EGFR signaling when β_H_ or AnxB9 is reduced is a result of elevated levels of EGFR signaling endosomes (see [5]). Thus, while mSpitz::GFP is a good marker for this compartment, there does not seem to be a conspicuous functional role for the capes in EGFR signaling.

## Discussion

**Figure 6:**
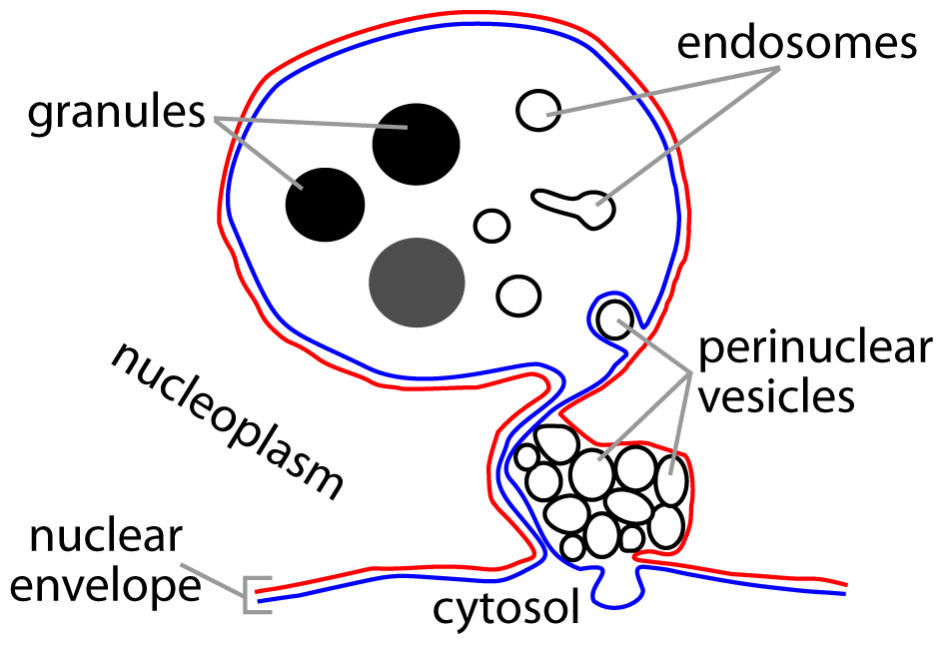
A generalized drawing of a cytoplasmic cape. All wild-type capes have two regions: (1) A complex area of membrane folding at the nuclear periphery that contains abundant perinuclear vesicles. These vesicles are bound by a single membrane and separated from the nucleoplasm by a single membrane bilayer; (2) A terminal chamber where the two membrane bilayers separate single membrane bound organelles and granules (with no obvious membrane layer) from the nucleoplasm. The universal juxtaposition of these two regions seen by extensive serial sectioning leads us to hypothesize that their formation and perhaps function is linked. Blue line – Outer nuclear membrane; Red line – inner nuclear membrane.

In this paper we probed the relationship between the membrane skeleton protein β_H_ and its partner AnxB9, and cytoplasmic capes- infoldings of the nuclear envelope. Figure 6 summarizes the structural features of a cytoplasmic cape based on our findings. We show that this subcompartment of the nuclear envelope accumulates large amounts of the EGFR ligand precursor mSpitz, is enriched in endosomal recycling proteins and are structurally altered in the absence of β_H_ or AnxB9. It is clear that cytoplasmic capes do not simply enclose bulk cytoplasm since we have identified several proteins that are present in membrane or terminal chamber of the capes, but also several that are not. We further show that a reduction in β_H_ does not result in a change in the amount of secreted Spitz ligand, despite causing elevated EGFR signaling. This suggests that the presence of the ligand precursor mSpitz in the capes is not obligatory for it's processing.

In recent years the role of spectrins has expanded from the structural lattice of the SBMS seen in the erythrocyte. In particular it has become evident that these giant proteins have important roles in endomembrane trafficking (e.g. [5]). Roles in DNA repair [8] and nuclear positioning [7] have also been reported. In this study we show that β_H_ also influences the development of cytoplasmic capes at the nuclear envelope. We were led to this discovery while further investigating our previously published genetic suppression of *rho^ve^* mutations (which down regulate EGFR signaling) by *karst* (β_H_) mutations. Although the EGFR ligand precursor mSpitz is in cytoplasmic capes along with a striking accumulation of endosomal markers, it seems that their perturbation by reductions in β_H_ does not influence the amount of processed sSpitz.

Just how β_H_ influences these structures is unclear and hard to determine since its wild-type levels in the cytoplasm are not readily detected. For a cytoplasmic cape to form, the nuclear envelope must bend away from the cytoplasm. Vertebrate AnxA1 is required for inwards vesiculation of intralumenal vesicles at the MVB [21] suggesting a possible mechanism for the role of AnxB9 in cape formation by facilitating membrane folding away from the cytoplasm. βH33, a C-terminal fragment of β_H_, causes this type of membrane extrusion in association with AnxB9 [5], [20]; however, we did not detect any influence on cape structure when we overexpressed βH33. The protein aPKC, which has been associated with PNV formation *via* phosphorylation of Lamin C [22], is part of the apical polarity complex that includes Crumbs, which recruits β_H_ to the apical membrane [23], [24]. So the influence of β_H_ on the capes may arise indirectly *via* modulation of the Crumbs complex or it's trafficking [19].

Cytoplasmic organelles have been seen in cytoplasmic capes [17], [25] and the discovery that multiple endosomal markers are present in cytoplasmic capes suggests both that endosomes are present in these structures and that protein sorting is occurring at this location. If this is true, then the concentration of mSpitz in cytoplasmic capes may have functional importance not detected by our assays. The processing of mSpitz to sSpitz is a complex process that begins in the ER when it becomes associated with the chaperone protein Star and moves to an endosome-related compartment [26]. In this compartment Rhomboid-1, an intramembrane protease, cuts mSpitz to release sSpitz, which is in turn secreted by exocytosis in a Rab11 dependent manner [10]. Complete details of this trafficking process have yet to be worked out and in particular direct visualization of Golgi transit, although inferred has only been seen in heterologous cell types [10], [12], [13], [15], [27]. Since mSpitz prominently accumulates in the capes, which contain the outer nuclear membrane but not other ER membranes, our data adds to this story by demonstration that mSpitz is accumulating very specifically on the outer nuclear membrane and not in more distant circum-nuclear ER. Recent data has shown that endosomes are always in very close contact with the ER, possibly facilitating cholesterol exchange and/or protein modifications [28]. In view of the close juxtaposition of the endosomal markers with the mSpitz in the cytoplasmic capes, we speculate that this could offer the opportunity for mSpitz to move directly from the ER to an endosomal compartment in these cape like structures and that the infolding of the nuclear membrane may protect the bulk ER from such activities.

It is also possible that the presence of endosomal markers in cytoplasmic capes indicates that these proteins are performing a distinct role at this location. The capes we describe were originally identified as sites of budding of the inner nuclear membrane to form vesicles in the perinuclear lumen (perinuclear vesicles) and of possible export of RNA to the cytoplasm [17], [25]. Recent confirmation of the latter notion has identified what appear to be the same structures as sites of a non-nuclear pore mRNA export pathway [22], and it has been speculated that this process is a normal manifestation of the nuclear egress pathway used by intranuclear viruses [29], [30]. While the presence of the C-terminal fragment of the Frizzled 2 receptor, which regulates RNP formation in muscle cells, in what looks like a Lamin C-positive cape in salivary glands [22] leads us to believe that the structures we have characterized will prove to be associated with RNP export, this has yet to be shown. Some of the original studies identifying cytoplasmic capes noted that these greatly increase in abundance in the mid to late third instar [17], [31], and it will be interesting to see if the assembly of these structures responds to ecdysone or other signaling molecules during larval development.

Another nuclear membrane invagination that can involve invagination of both the INM and ONM is the nucleoplasmic reticulum (reviewed in [32]). This is seen in many mammalian tissue culture cell nuclei, but is greatly exaggerated in certain laminopathy-associated mutant backgrounds. Nucleoplasmic reticulum invaginations appear to be distinct from cytoplasmic capes since they lack associated regions of PNV, often appear to be more sheet-like in morphology and can be conspicuously branched. Furthermore, the nucleoplasmic reticulum contains cytoplasmic organelles such as mitochondria (*ibid*) that we have not seen encapsulated in any cape. Whether these will prove to be a species specific variant on the cytoplasmic cape must await future investigations.

Finally, there do appear to be nuclear roles for endosomal proteins [33], [34], and since budding of the PNV is topologically similar to intralumenal vesicle formation at the MVB it is possible that similar machinery may be involved. How this pathway might interact with a potential protein exit route from the ER to endosomes at this location remains unclear at this time.

## Supporting Information

Figure S1 **A selection of compartment markers not found in cytoplasmic capes.** Several markers we have examined are not found in cytoplasmic capes suggesting that there may be specificity in the cytoplasmic organelles which enter this region. Six examples are shown here. All GFP/YFP construct are driven by AB1-Gal4. **A** – Staining for Lava lamp, a Golgi marker; **B** – 2xFYVE::GFP marking PtdIns(3)P positive compartments; **C** – RabX1::YFP; **D** – Rab4::YFP; **E** – Rab7::YFP; **F** – Rab10::YFP.
(TIF)

Figure S2 **β_H_ overexpression but not knockdown perturbs EGFR distribution in the salivary gland.** **A-A”** – Staining for EGFR in early, mid and late third instar salivary glands (Note the progressive appearance of secretory granules). EGFR steadily declines. **B** – When β_H_ is knocked down (AB1>*kst^RNAi^*) the distribution of EGFR does not change. However, the observation that the receptor is gradually lost from the apical membrane during the third instar (A-A”) suggests that its *de novo* synthesis and turnover is likely to be low at this stage in development. In vertebrates, treating cells with high levels of EGF will drive internalization of EGFR *via* large tubular-vesicular structures (Sorkin and Goh, 2008). To achieve a similar situation and accumulate the receptor in internal compartments we overexpressed wild-type EGFR in the salivary gland. Panels C–D” show salivary glands overexpressing EGFR (AB1>EGFR) stained for β_H_ (left panels) and EGFR or EPS15 (right panels). Central panel shows a merged image with β_H_ in green. **C-C”** – β_H_ and EGFR are both found on internal vesicles that cluster and partially colocalize (Arrows). **D-D”** – β_H_ and EPS15 are both found on internal vesicles that cluster and partially colocalize (Arrows). Note that a second dispersed population of EPS15 puncta remains throughout the cytoplasm. EGFR was never detected in the perinuclear region.
(TIF)

Movie S1 **A sequence of sections derived by SBF-SEM through about one half of a nuclear diameter.** Capes are indicated by dashed arc lines as they emerge with coloured arrows indicating the terminal chambers. Note how all capes are associated with terminal chambers.
(MOV)

Movie S2 **An enlarged sequence of a cape seen in Movie S1.** The origin at the nuclear membrane is evident about half way through the sequence. A large terminal chamber with some internal vesicles emerges in the last third of the sequence.
(MOV)

Movie S3 **A sequence of images taken for electron tomography illustrates the 3D structure of a 250 nm slice through a cape.** Visible in this section are parts of both the terminal chamber and the perinuclear vesicle (PNV) regions. Red arrows show where the double membrane layer surrounding the terminal chamber is clearly visible. Blue arrows point to examples of membrane bound vesicle present in this chamber.
(MOV)
