# Supplementary figures and images for: Cytoplasmic Capes Are Nuclear Envelope Intrusions That Are Enriched in Endosomal Proteins and Depend upon βH-Spectrin and Annexin B9
Source: PLoS One. 2014 Apr 4;9(4):e93680. doi: 10.1371/journal.pone.0093680 (PMC3976414; doi:10.1371/journal.pone.0093680)

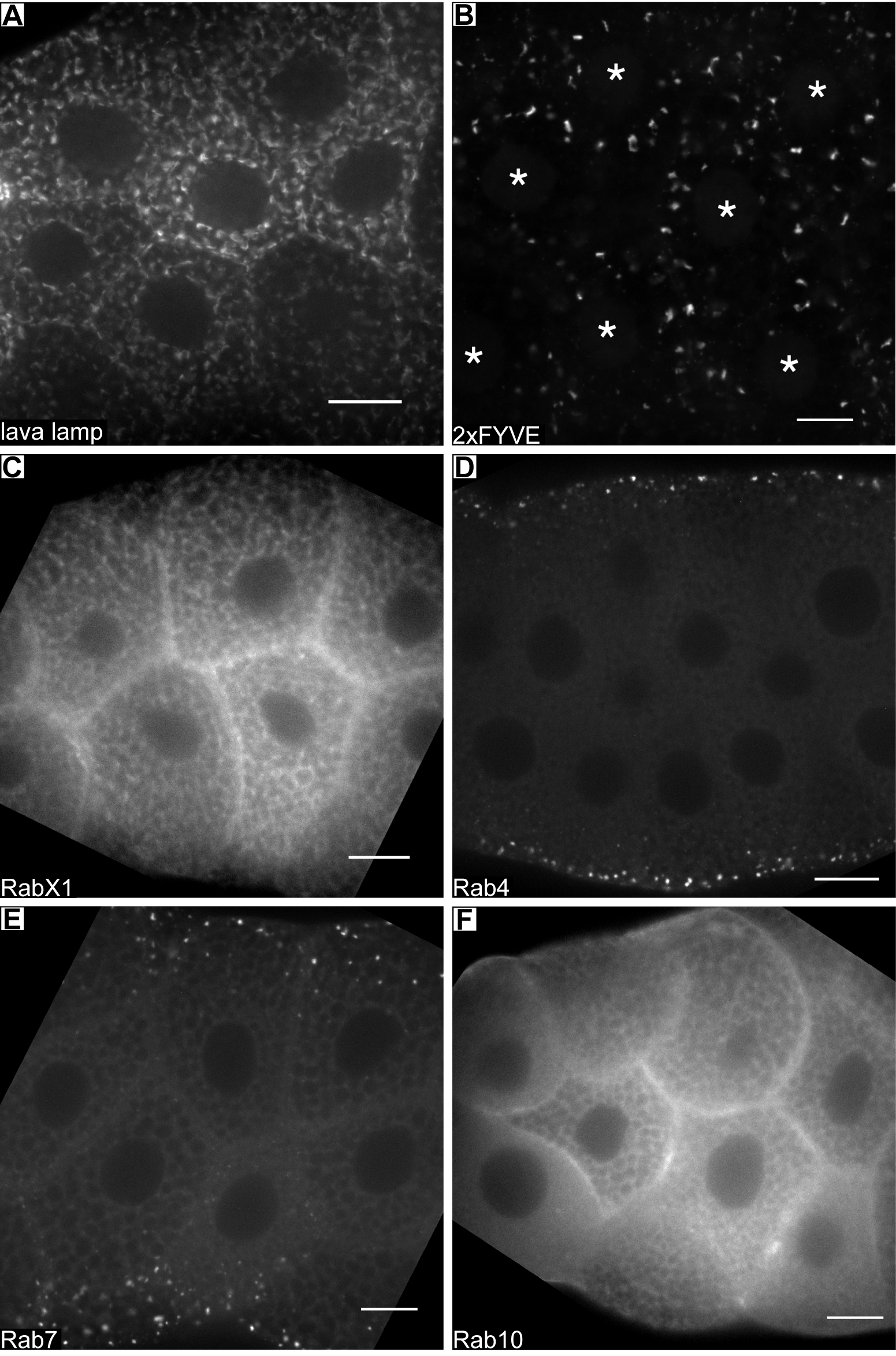

Supplement: Figure S1 — A selection of compartment markers not found in cytoplasmic capes. Several markers we have examined are not found in cytoplasmic capes suggesting that there may be specificity in the cytoplasmic organelles which enter this region. Six examples are shown here. All GFP/YFP construct are driven by AB1-Gal4. A – Staining for Lava lamp, a Golgi marker; B – 2xFYVE::GFP marking PtdIns(3)P positive compartments; C – RabX1::YFP; D – Rab4::YFP; E – Rab7::YFP; F – Rab10::YFP. (TIF) [file pone.0093680.s001.tif]

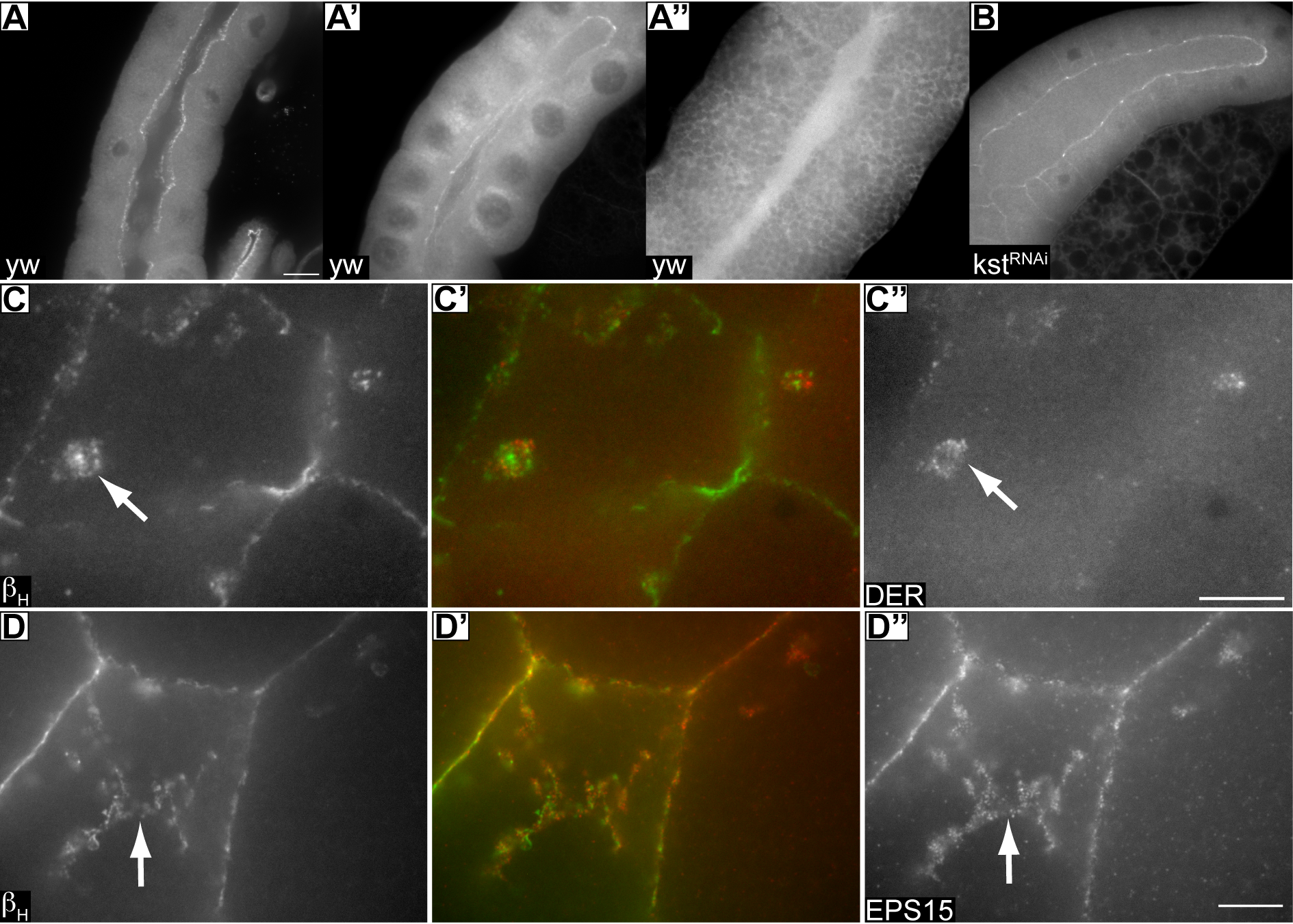

Supplement: Figure S2 — βH overexpression but not knockdown perturbs EGFR distribution in the salivary gland. A-A” – Staining for EGFR in early, mid and late third instar salivary glands (Note the progressive appearance of secretory granules). EGFR steadily declines. B – When βH is knocked down (AB1>kstRNAi) the distribution of EGFR does not change. However, the observation that the receptor is gradually lost from the apical membrane during the third instar (A-A”) suggests that its de novo synthesis and turnover is likely to be low at this stage in development. In vertebrates, treating cells with high levels of EGF will drive internalization of EGFR via large tubular-vesicular structures (Sorkin and Goh, 2008). To achieve a similar situation and accumulate the receptor in internal compartments we overexpressed wild-type EGFR in the salivary gland. Panels C–D” show salivary glands overexpressing EGFR (AB1>EGFR) stained for βH (left panels) and EGFR or EPS15 (right panels). Central panel shows a merged image with βH in green. C-C” – βH and EGFR are both found on internal vesicles that cluster and partially colocalize (Arrows). D-D” – βH and EPS15 are both found on internal vesicles that cluster and partially colocalize (Arrows). Note that a second dispersed population of EPS15 puncta remains throughout the cytoplasm. EGFR was never detected in the perinuclear region. (TIF) [file pone.0093680.s002.tif]
